# Accessory splenic infarction presenting as acute abdominal pain: A case report

**DOI:** 10.1016/j.ijscr.2025.110852

**Published:** 2025-01-06

**Authors:** Dana Awad, Danielle Abou Khater, Peter Habchy, Mariana Helou

**Affiliations:** a Emergency Medicine Department, Lebanese American University Medical Center, Beirut, Lebanon; b General Surgery Department, Lebanese American University Medical Center, Beirut, Lebanon

**Keywords:** Accessory spleen, Ectopic splenic tissue, Infarction, Splenule, Abdominal pain

## Abstract

**Introduction:**

Accessory spleens are a common anatomical variant, consisting of ectopic splenic tissue present in different locations in the peritoneal cavity. Typically asymptomatic, the presence of these tissue grows to be of clinical importance when complicated by infarction, rupture, or torsion.

**Presentation of case:**

We report the case of a 36-year-old female that presented to the Emergency Department for diffuse abdominal pain and was found to have a partially ruptured splenule secondary to a venous infarct on abdominal computed tomography scan. The patient was admitted to the hospital for pain management and further workup. Her hospital stay was uncomplicated with complete resolution of symptoms after 5 days.

**Discussion:**

The usually asymptomatic accessory spleen can present in case of infarction with vague symptoms like abdominal pain, nausea, or vomiting. It is triggered by conditions such hematologic disorders, embolic disorders, vascular disorders, and trauma. Oral contraceptive pills increase thrombosis risk by affecting coagulation factors, making them a potential cause of infarction. Diagnosis typically involves CT imaging, and treatment ranges from supportive care to surgical intervention.

**Conclusion:**

Accessory spleen infarction, although rare, is a diagnosis that should be considered in the assessment of a patient presenting to the emergency with acute abdominal pain.

## Highlights

• An accessory spleen known as splenule or splenunculus is a benign ectopic splenic tissue that can be found in up to 30% of the population as a result from failure of the splenic anlage fusion during embryology.
• Most accessory spleens are usually asymptomatic but can present with acute complications like torsion, infarction, or rupture.
• Accessory spleen torsion occurs when venous return is compromised, causing hemorrhagic infarction. It can present as abdominal pain, nausea, vomiting, or an acute abdomen.
• Diagnosis relies on abdominal computed tomography, showing hypodense areas in the spleen.
• Oral contraceptive pills (OCPs) significantly increase the risk of thrombosis by altering coagulation factors, which can lead to splenic infarction, as seen in reported cases of accessory spleen infarction linked to OCP use.

## 1 Introduction

An accessory spleen, also known as a splenule or a splenunculus, is a benign ectopic splenic tissue present congenitally as a result of the failure of the splenic anlage to fuse during embryology [1]. They are a relatively common finding, with autopsy studies showing an incidence of 10–30 % in the general population [2,3]. An accessory spleen can be distinguished from other forms of ectopic splenic tissue, such as splenosis, by the fact that an accessory spleen has the same functionality and histology as a spleen congenitally. In contrast, splenosis results from the detachment of a part of the spleen due to trauma and its embedment in an ectopic location in the peritoneal cavity, followed by recruitment of nearby bloody supply [4]. About 75 % of accessory spleens are found at the splenic hilum, but they can also appear along the pancreatic border, in the splenogastric omentum, greater omentum, pelvis, or anywhere else in the abdomen [5,6]. The presence of an accessory spleen is predominantly asymptomatic; however, certain complications such as infarction, torsion, and rupture may occur necessitating medical and surgical interventions. We report the case of a young female presenting to the Emergency Department (ED) for acute abdominal pain caused by an infarcted and partially ruptured accessory spleen. All work has been reported in line with the SCARE criteria [7].

## 2 Case presentation

A 36-year-old female presented to the ED for a 3-day history of diffuse abdominal pain associated with nausea and an episode of non-bloody, non-mucoid diarrhea. The abdominal pain started in the left upper quadrant (LUQ) then became diffuse with worsening intensity over the span of 2 days. Over the counter analgesics and antispasmodics did not improve the pain. She denied any fever, vomiting, blunt abdominal trauma, or recent miscarriages.

The patient is a light smoker, consuming approximately three cigarettes per day for the year, with a past medical history of thalassemia minor and a surgical history of appendectomy. Her home medications include Combination Oral contraceptives (OCP) once daily. The OCP was prescribed for the purpose of contraception as well as regulation of the patient's menstrual cycle and has been used by the patient for the past 17 years.

Upon presentation, the patient was afebrile with normal vital signs. Physical examination was pertinent for severe diffuse abdominal tenderness, left upper quadrant guarding and bilateral lower quadrant rebound tenderness indicative of regional peritonitis.

Laboratory values revealed a white blood cell count of 9800 cells per microliters and an elevated C-Reactive Protein of 108 mg/L. Her hemoglobin and creatinine were within normal limits, alongside her liver enzymes and lipase levels.

**Fig. 1:**
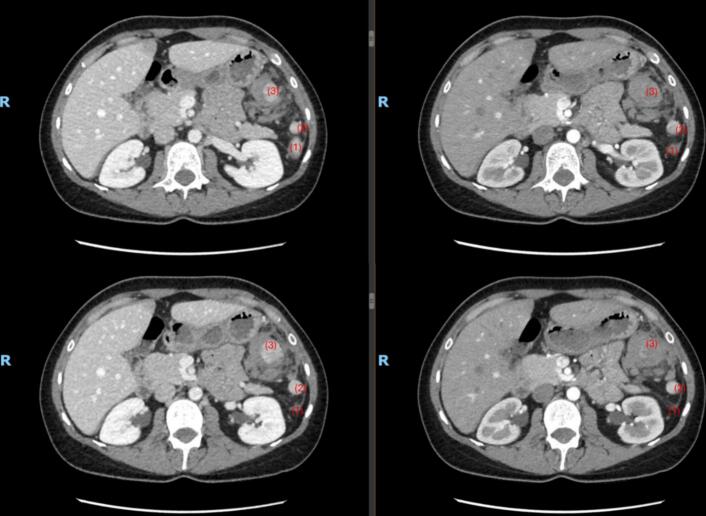
Axial cuts of the abdominal CT scan with contrast injection done upon presentation: portal phase on the left side and arterial phase on the right side. (1) Represent the caudal main spleen, (2) represent the unremarkable accessory spleen, (3) represent the ruptured accessory spleen with surrounding pooling hematoma.

Enhanced computed tomography (CT) scan revealed a partial rupture of a splenule in the LUQ, possibly to a venous thrombosis at the distal splenic vein with surrounding hematoma and evidence of a moderate amount of hemoperitoneum. No radiological evidence suggestive of an active bleed (Fig. 1).

Based on these findings, surgical and hematological consultations were done. As part of the evaluation and treatment plan, the patient's hypercoagulable profile was assessed, and she was placed on pain management and supportive care. Additionally, the patient was educated about the importance of smoking cessation while on oral contraceptives and their relation to increasing her thrombotic risk.

**Fig. 2:**
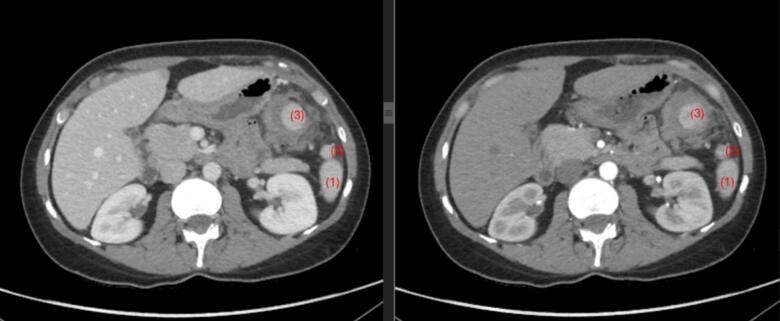
Axial cuts of the follow up abdominal CT scan with contrast injection: portal phase on the left side and arterial phase on the right side. (1) Represent the caudal main spleen, (2) represent the unremarkable accessory spleen, (3) represent the ruptured accessory spleen with interval further decrease in the surrounding evolving hematoma.

Follow up enhanced CT scan performed 5 days after admission revealed an increase in the size of the accessory spleen's core, and a resolving hemoperitoneum (Fig. 2). These findings were in keeping with a favorable resolving process. She had an uneventful 5-day hospital stay after which all consultants agreed on discharge on pain management.

## 3 Discussion

Accessory spleens lead a relatively silent clinical course yet certain acute complications such as infarctions, torsions, and ruptures may occur leading to a clinical presentation of acute abdominal pain. Acute accessory spleen torsion occurs when the venous return to the ectopic splenic tissue is compromised leading to a hemorrhagic infarction of parenchyma [8]. Presenting symptoms are not specific and may range from abdominal pain, nausea, vomiting to an acute abdomen. In certain cases, the clinical presentation may include intermitted recurrent abdominal pain which could be due to intermittent self-resolving torsions [9].

Etiologies causing accessory spleen infarcts are similar to those causing spleen infarcts and can be categorized into malignant hematologic disorders, benign hematologic disorders, embolic disorders, vascular disorders, and trauma [10,11]. The diagnosis of a splenic injury should be initiated with high clinical suspicion. The diagnostic method of choice is an enhanced abdominal computed tomography characteristically showing wedge-shaped, linear, or peripheral round hypodense areas in the spleen [11]. In the emergency setting however, and in the case of a hemodynamically unstable patient, a bedside ultrasound can allow for the early detection of hemoperitoneum [10].

Oral contraceptive pills increase the relative risk of thrombosis by three to five-fold, with the risk depending on the estrogen dose, type of progestin, mechanism of delivery, and length of therapy [12]. Estrogen affects hemostasis by raising the levels of clotting factors (VII, VIII, X, and fibrinogen) and plasminogen, while reducing levels of antithrombin III and protein S, and altering resistance to activated protein C. Activated protein C is a zymogen that inactivates factor Va; when resistance to activated protein C increases, this inhibition does not occur, allowing the coagulation cascade to continue. As a result, combination pills tend to have a procoagulant effect [13]. Al-Ola et al. [14] reported a case of accessory splenic infarction in a previously healthy female on OCP. Similarly, Arul et al. [15] reported a case of splenic infarction secondary to celiac thrombosis in a young female on OCP. Additionally, smoking increases the thrombotic risk in the female population in comparison to the male population, and there exists a synergistic effect between smoking and oral contraceptive use in increasing the risk of thrombosis [16]. Tzankova et al. reported that in women who are smokers and on OCPs the risk of venous thromboembolisms and their complications, such as pulmonary embolisms, are substantially increased [17].

An extensive workup is recommended to reveal the etiology causing an accessory spleen infarction. In the case discussed above, coagulation studies were within the reference range, and the patient had no past medical history suggestive of an underlying disorder that could be at the base of the diagnosed infarct. The patient's long-standing use of a combination oral contraceptive pill is hypothesized to be the major predisposing factor. Regarding its management, treatment of a splenic infarct depends on the underlying etiology and ranges from supportive treatment with pain medications and IV hydration, to urgent surgical interventions in the cases of hemodynamic instability [10].

## 4 Conclusion

Accessory splenic infarct, although rare, should be considered as a potential diagnosis in patients who present with sudden left upper quadrant abdominal pain. Its clinical presentation can mimic other acute abdominal conditions such as appendicitis, pancreatitis, or bowel ischemia, rendering its diagnosis challenging. The accessory spleen in this case was an incidental finding only later detected on imaging.

## Author contribution

**Dana Awad**: Lead Author, responsible for drafting the manuscript and overseeing its revisions.

**Danielle Abou Khater**: Manuscript Editor Co-Author, responsible for reviewing and refining the manuscript.Also acting as the primary contact with the journal throughout the submission and review process.

**Peter Habchy**: Literature Review Co-Author, tasked with conducting a thorough literature review to ensure the case report is well-situated within current medical knowledge.

**Mariana Helou**: Senior Specialist Author, providing supervision and critical revisions to ensure the manuscript accurately represents the case.

## Consent

Informed consent was obtained from the patient for publication.

## Ethical approval

Ethics approval is not required for this case report as it solely focuses on a descriptive analysis of an individual case, without any modifications to the standard clinical management or additional risk to the patient.

## Guarantor

Dr Mariana Helou.

## Research registration number

Not applicable.

## Funding

The authors of this study declare that there are no funding sources.

## Conflict of interest statement

No conflicts of interest to declare.
